# A Novel Method for Vertical Acceleration Noise Suppression of a Thrust-Vectored VTOL UAV

**DOI:** 10.3390/s16122054

**Published:** 2016-12-02

**Authors:** Huanyu Li, Linfeng Wu, Yingjie Li, Chunwen Li, Hangyu Li

**Affiliations:** 1 Department of Automation, Tsinghua University, Beijing 100084, China; lihuanyu12@mails.tsinghua.edu.cn (H.L.); wulf13@mails.tsinghua.edu.cn (L.W.); liyj08@mails.tsinghua.edu.cn (Y.L.); lcw@mail.tsinghua.edu.cn (C.L.); 2 Department of Energy Resources Engineering, Stanford University, Stanford, CA 94305, USA

**Keywords:** data fusion, noise suppression, Kalman filter, acceleration, VTOL UAV, thrust-vectoring

## Abstract

Acceleration is of great importance in motion control for unmanned aerial vehicles (UAVs), especially during the takeoff and landing stages. However, the measured acceleration is inevitably polluted by severe noise. Therefore, a proper noise suppression procedure is required. This paper presents a novel method to reduce the noise in the measured vertical acceleration for a thrust-vectored tail-sitter vertical takeoff and landing (VTOL) UAV. In the new procedure, a Kalman filter is first applied to estimate the UAV mass by using the information in the vertical thrust and measured acceleration. The UAV mass is then used to compute an estimate of UAV vertical acceleration. The estimated acceleration is finally fused with the measured acceleration to obtain the minimum variance estimate of vertical acceleration. By doing this, the new approach incorporates the thrust information into the acceleration estimate. The method is applied to the data measured in a VTOL UAV takeoff experiment. Two other denoising approaches developed by former researchers are also tested for comparison. The results demonstrate that the new method is able to suppress the acceleration noise substantially. It also maintains the real-time performance in the final estimated acceleration, which is not seen in the former denoising approaches. The acceleration treated with the new method can be readily used in the motion control applications for UAVs to achieve improved accuracy.

## 1. Introduction

Vertical takeoff and landing (VTOL) unmanned aerial vehicles (UAV) have attracted the attention of many researchers [1,2]. For VTOL UAVs, the vertical motion control design is of great importance, especially during the takeoff and landing stages [3,4]. This is because a motion control failure can result in severe damage to the UAV. To achieve well-performed vertical motion control, high-quality vertical acceleration feedback should be used because it enables the controllers to take action before the unexpected dynamic manifests itself in velocity and height [5,6,7].

However, the acceleration measurement can be severely polluted by noise in real applications, which is a major challenge of acceleration feedback control [8]. A lot of research has been done to reduce the acceleration noise.

Sun et al. developed an infinite impulse response (IIR) digital lowpass filter to remove the high frequency noise in acceleration measurement [9]. The parameters of the lowpass filter were adopted according to the characteristics of the acceleration noise. An experiment was conducted to verify the performance of the lowpass filter. Because of its simplicity, a lowpass filter has been widely used in denoising applications [10]. Lu et al. developed a procedure which consists of an IIR lowpass filter and a Kalman filter to attenuate the influence of sensor noise and external disturbance [11]. The lowpass filter is applied at first to eliminate the high frequency noise of the measured acceleration. The Kalman filter is then used to further reduce the noise. The noise reduction performance was verified by experiment. The standard deviation of acceleration noise was reduced from 0.33 m/s${}^{2}$ to 0.02 m/s${}^{2}$. In addition, El-Sheimy et al. introduced the wavelet filter to reduce the acceleration noise [12]. The six-level wavelet decomposition is used to eliminate the high frequency noise from the low frequency signal. A significant improvement in the quality of the acceleration signal was achieved. The standard deviation of the acceleration noise was reduced by about 99%.

Though the above methods are able to reduce acceleration noise effectively, these studies were all taken in a constrained environment in which the acceleration is very stable with only small variations. For acceleration, which varies significantly with time, the noise reduction becomes much more challenging. Kownacki investigated such problems using the Kalman filter, though the model was built with the assumption of constant acceleration [13]. A conflict between the output signal noise level and filter response rate was discussed. This discussion indicates that the filter response rate has to be sacrificed in order to achieve effective noise suppression performance. In fact, this dilemma also exists in all of the above noise reduction methods. Hebbale and Ghoneim exploited constant jerk (the derivative of acceleration) model in the design of a Kalman filter to reduce the acceleration noise [14]. Nevertheless, this method also suffers from the time delay just like the other approaches.

The above-mentioned methods have been used for UAV acceleration noise reduction. For example, Abellanosa et al. used lowpass filter to suppress the acceleration noise for position estimation of a Quadcopter UAV [15]. Quadri et al. applied a Kalman filter to reduce acceleration noise for attitude estimation of a multi-rotor UAV [16]. Related work can also be found in [17,18]. In those studies, the accelerations were mainly used for position and attitude estimation for UAVs. The presence of time delay in the existing denoising methods prevents the usage of acceleration feedback control for UAVs.

To overcome the problems in the previous denoising methods, we develop a new frame for acceleration noise suppression of a thrust-vectored UAV. Instead of the direct filter used in previous work, the new procedure combines the thrust information with the accelerometer measurement to obtain a more accurate acceleration estimate. The new method is able to suppress the acceleration noise without introducing time delay. A thrust-vectored tail-sitter UAV takeoff experiment is conducted to verify the effectiveness of our method. To the best of our knowledge, this is the first attempt to estimate UAV acceleration in this way.

This paper is organized as follows. In Section 2, the configuration of the thrust-vectored VTOL UAV and the experiment platform is introduced. Section 3 presents the detailed analysis of the statistical characteristics of the acceleration noise, which is the prerequisite for the noise suppression approach. In Section 4, we build an engine thrust model and a thrust vector model based on ground experiments which are used to calculate the vertical thrust when UAV is flying. The noise reduction procedure is then described in Section 5, which includes a Kalman filter to estimate UAV mass and a data fusion step to obtain the minimum variance estimate of acceleration. In Section 6, we present the results for the UAV takeoff experiment. Two existing methods are tested as well for comparison purpose. A section to summarize the work is finally presented.

## 2. UAV Configuration and Experiment Platform

**Figure 1:**
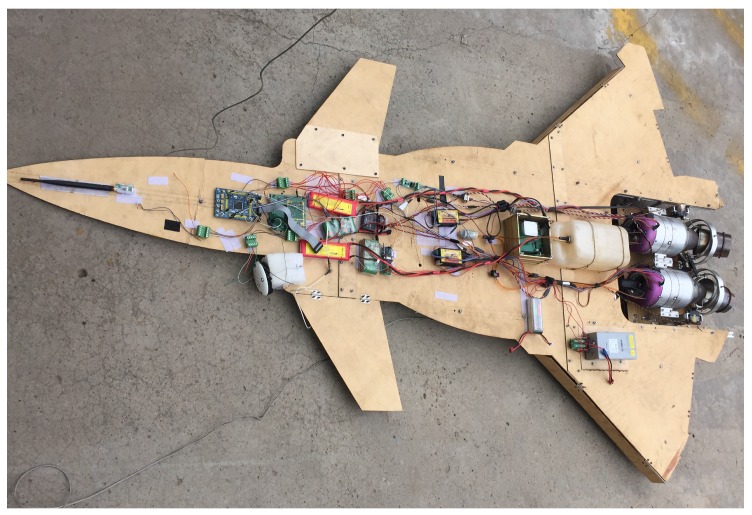
The thrust-vectored tail-sitter unmanned aerial vehicle (UAV).

Tsinghua University has developed a new prototype tail-sitter VTOL UAV, which is equipped with a thrust-vectoring engine system as shown in Figure 1. The UAV has a length of 2 m, a wingspan of 1.6 m, and the gross weight of the UAV (including the thrust vectoring system) is about 24 kg. The thrust vectoring system includes two micro turbine engines with independent vectoring nozzles. The turbine engine used is Jetcat P200. P200 is a single-spool axial flow turbine engine with a net weight of 2.4 kg and a maximum thrust of 230 N which corresponds to a maximum rotor speed of 112,000 revolutions per minute (RPM). With two turbine engines, this UAV has a maximum thrust of 460 N which enables an approximately 46 kg maximum takeoff mass. The detailed technical parameters of the engine are available in [19].

The electronic system of the UAV includes a microcomputer, an inertial measurement unit (IMU), a Hall sensor, a laser range finder and other auxiliary communication and measurement instruments. The microcomputer board with STM32F407 microprocessor is used to perform data processing and give commands to the thrust vectoring system in order to keep the UAV stable. The IMU (ADIS16488A from Analog Devices) provides the attitude and acceleration information to the microcomputer with a sample frequency of 25 Hz. It is installed in the center of gravity of the UAV to prevent the rotation of the UAV body from disturbing acceleration measurement. The Hall sensor is used to measure the rotor speed with very high accuracy (±10 RPM for rotor speed at over 85,000 RPM). The laser range finder (DLSB-15 from Dimetix) with a sample frequency of 5 HZ and a measurement error within ±1.5 mm is used to measure the height from the ground for the UAV.

**Figure 2:**
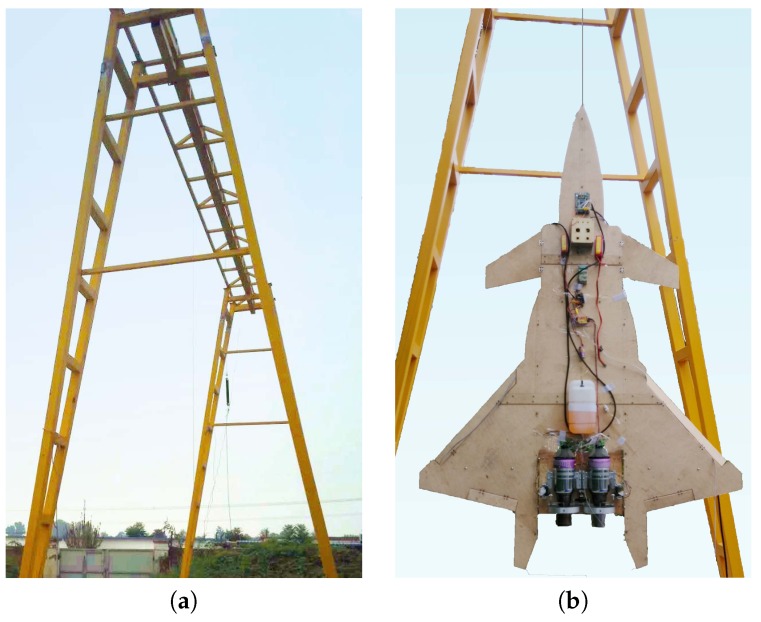
The experiment platform. (**a**) Gantry crane; (**b**) Suspended UAV.

To ensure the safety of the experiment (for both the staff and UAV body) and also for the convenience of the experiment, the UAV does not directly take off and land on the ground. Similarly to the UAV VTOL experiment study in [20], it is hung with a cable under a gantry crane which is shown in Figure 2a. The height of the gantry crane is 7.2 m, and the width is 6.8 m. To avoid the ground effect influencing the thrust performance, the tail of the UAV is hung at about 1.5 m high. As mentioned previously the UAV itself has a length of 2 m, therefore the operating window for the UAV is about 3.5 m. During the experiment, the UAV height is monitored closely to ensure it does not hit the crane. The UAV hanging under the crane is shown in Figure 2b. The experiment is conducted in calm days with wind speed less than 1 km/h to minimize the external disturbance.

## 3. Analysis of Acceleration Noise

The acceleration measured with IMU is inevitably polluted by noise. The noise can be caused by many reasons, for example, the external disturbances (such as wind), and the vibration of the turbine engines. To develop a noise reduction procedure, the characteristics of the noise need firstly to be investigated.

In this work, only the vertical acceleration is analyzed because it is of greater importance for UAV motion control. The vertical acceleration is not directly measured in the strap-down inertial system. Instead, the acceleration is measured in the UAV body coordinate system. This measured acceleration needs to be transformed from the UAV body coordinate system to the inertial coordinate system. Previous work on coordinate transformation for tail-sitter UAVs has been done by other researchers [21]. It is directly used in this paper without repetitive description. In this work, all the acceleration data presented is in the inertial coordinate system (after transformation).

**Figure 3:**
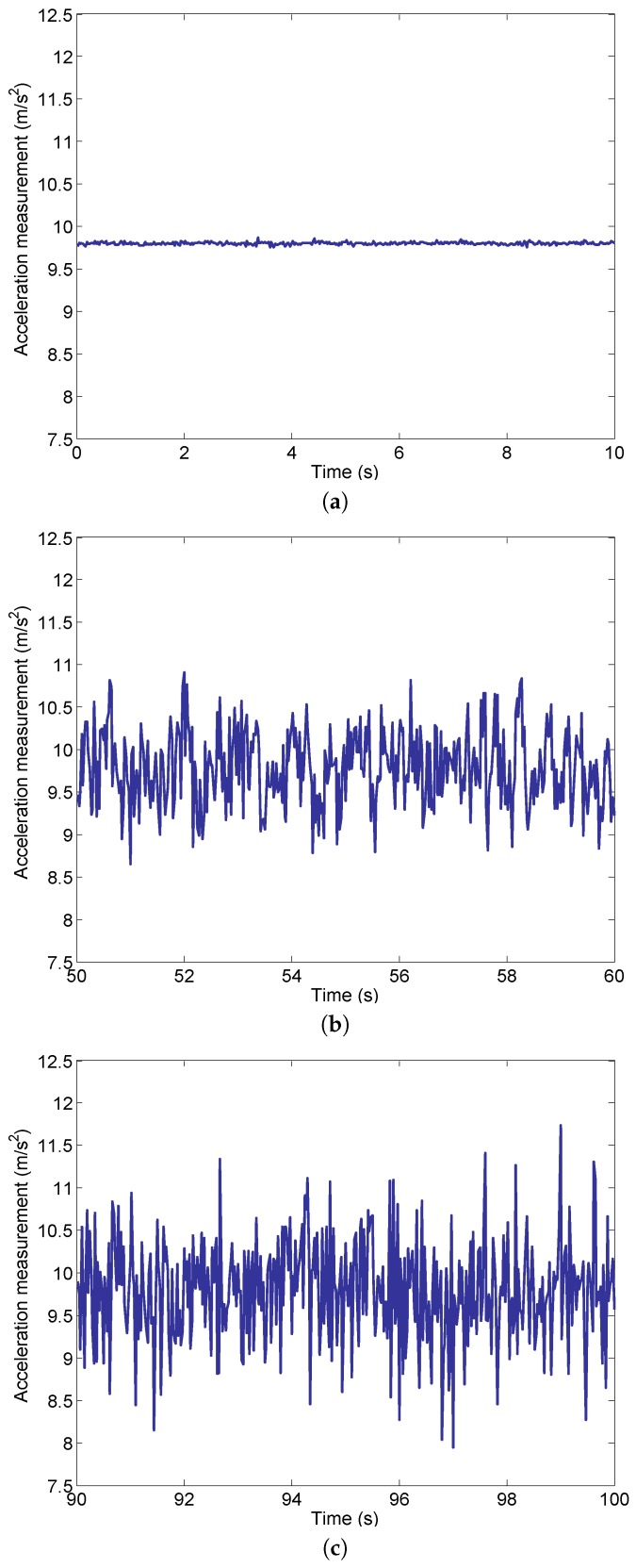
Vertical acceleration measurements at different engine states. (**a**) Vertical acceleration measurement before the engines start; (**b**) Vertical acceleration measurement when the engines work at idle (33,000 RPM); (**c**) Vertical acceleration measurement when the engines work close to takeoff (85,000 RPM).

The measurements of UAV vertical acceleration at three different engine states are shown in Figure 3. Figure 3a shows the acceleration before the engines start, Figure 3b shows the acceleration when the engines work at an idle speed (33,000 RPM), while Figure 3c shows the acceleration when the engines work close to the takeoff speed (85,000 RPM). It should be noted that the engine system does not reach the takeoff thrust at the rotor speed of 85,000 RPM, therefore the UAV remains stationary for all three states.

Because the IMU measures the proper acceleration (physical acceleration) instead of coordinate acceleration (rate of change of velocity), the measured acceleration should be the gravitational acceleration when the UAV stays stationary. As shown in Figure 3a the acceleration measurement is very close to 9.8 m/s${}^{2}$ with small-scale noise. In contrast, the acceleration noise becomes more significant after the engines start and it grows as the rotor speed increases from 33,000 to 85,000 RPM. This is because of the vibration in the engines due to the rotation of the rotor and the disturbance of the gas flow. Note that the rotor speed of 85,000 RPM is close to the critical rotor speed for UAV takeoff.

The mean and variance for each of the three acceleration measurements are shown in Table 1. It can be seen that the mean values are very close to each other. The slight difference between them is because of the finite amount of data measured during the experiments. The largest difference is only 0.009 m/s${}^{2}$, which is negligible compared with the gravitational acceleration. In this work, gravitational acceleration is adopted as 9.797 m/s${}^{2}$ which is the average of the three mean values in Table 1. In contrast, the computed variance values differ from each other significantly. When engines are shut down (before start), the variance is very small and it increases dramatically when engine rotor speed is of 33,000 RPM or 85,000 RPM. This is consistent with the observation in Figure 3, since the variance in acceleration represents the intensity of the noise.

Many filtration and data fusion algorithms have intrinsic assumptions on the statistical characteristics of the noise. More detailed investigation of the noise is therefore needed. In this work, the acceleration noise in measured data is defined as:

(1) $$w=a_{m}-{\bar{a}}_{m}$$

where *w* represents the noise, $a_{m}$ is the measured acceleration and ${\bar{a}}_{m}$ is the mean of the measured acceleration.

We first compute the normalized autocorrelation, designated R(k), of the acceleration noise using Equation (2):

(2) $$R\left(k\right)=\frac{1}{N\sigma^{2}}\sum_{i=1}^{N}w\left(i\right)w\left(i+k\right)$$

where *N* is the number of sampled data points, $\sigma^{2}$ is the variance of the noise, w(i) and w(i+k) are the noise values at sampled data points *i* and i+k respectively, and *k* is the lag of the autocorrelation. The acceleration noise for engine rotor speed of 85,000 RPM (as shown in Figure 3c) is used for analysis because it corresponds to a state of greater importance (near the takeoff speed).

**Figure 4:**
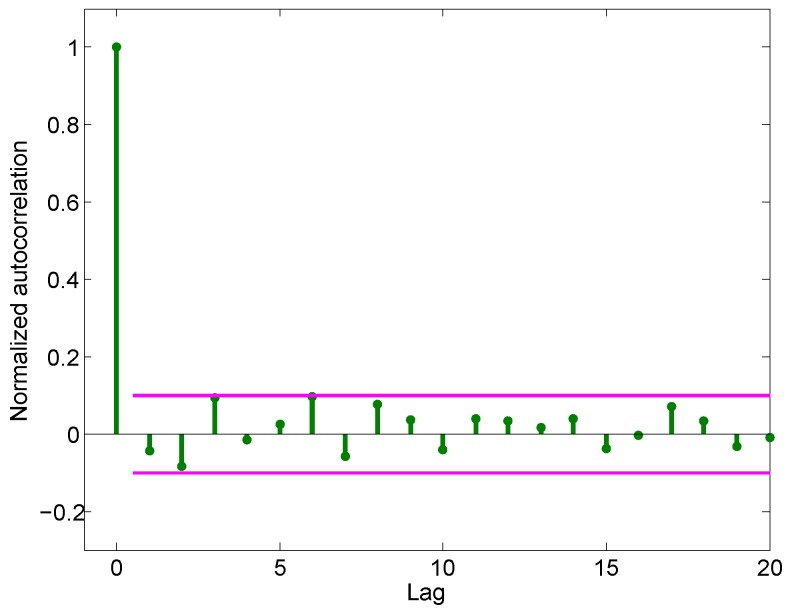
Normalized autocorrelation of the noise in measured acceleration.

The result of Equation (2) is shown in Figure 4 with the *x*-axis indicating the lag and the *y*-axis indicating the normalized autocorrelation of the sampled data. According to Figure 4, the autocorrelation of the acceleration noise can be regarded as an impulse function with high fidelity, which indicates that the acceleration noise can be treated as white noise.

**Figure 5:**
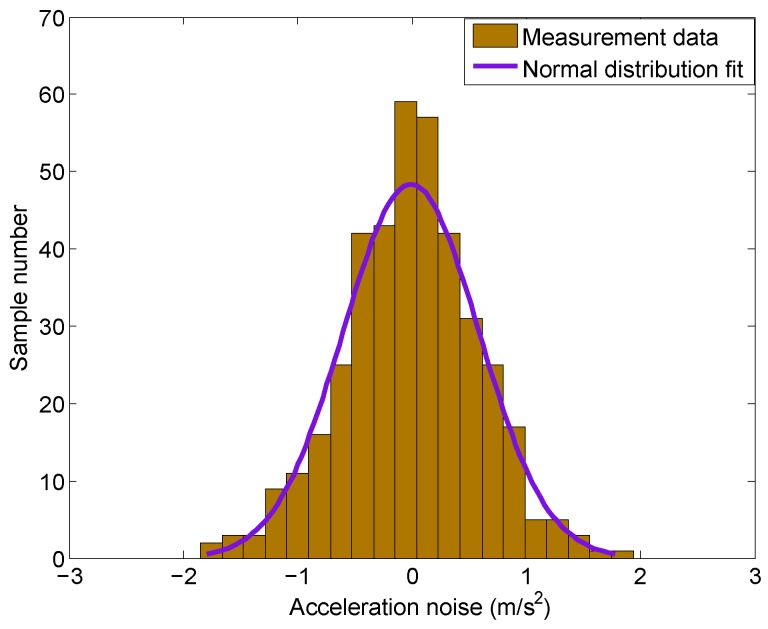
Histogram of the acceleration noise and normal distribution curve fit.

In addition, Figure 5 shows the histogram of the acceleration noise with a normal distribution curve fit (purple line). It can be seen that the normal distribution fits the experimental results very well, which suggests that the noise is of the Gaussian type.

In addition to the above analysis, Woodman [22] and Titterton [23] also stated that it is standard practice to assume that random errors follow a Gaussian distribution when modelling errors in acceleration measurement. Therefore, it is reasonable to assume the accelerometer measurement noise is a white Gaussian noise. This assumption is widely used in the literature, for example, in [11,13,16] mentioned before. The same assumption is used in this work as well.

## 4. Modeling of Engine Thrust System

During the takeoff and landing stages, the aerodynamic forces can be ignored because of the low air speed relative to the UAV. The engine thrust is the only non-gravitational force exerts on the UAV. The UAV motion dynamics in the vertical direction can therefore be written as:

(3) $$F_{v}-mg=ma$$

where $F_{v}$ denotes the vertical thrust of the engine system, *m* is the mass of the UAV, *g* is the gravitational acceleration, and *a* is the vertical coordinate acceleration with the positive direction upward. By introducing the proper acceleration $a_{p}$, Equation (3) can be rewritten as:

(4) $$a_{p}=g+a=F_{v}/m$$

This proportional relationship (Newton’s Second Law) between the proper acceleration and the vertical thrust is demonstrated in Equation (4). It inspires us to incorporate the thrust information into the acceleration estimation. However, because the engine thrust cannot be directly measured during flight, mathematical models are needed to calculate the thrust. In this section, we develop models to compute the thrust and thrust deflection angle based on measurements that can be obtained when the UAV is flying.

Note that the noise reduction method developed in this work (presented in Section 5) is independent of the engine thrust system model built here. The method can be readily used to reduce acceleration noise for other types of UAV platforms with different engines for which different thrust system models are needed. The thrust system model described in this section is for Jetcat P200 turbine engine used in our UAV platform.

### 4.1. Engine Thrust Model

Previous work has demonstrated that a single input single output model can be used to characterize the relationship between the thrust and rotor speed for micro turbine engines [24]. In this work, a similar relationship is built to model the P200 turbine engine. Since the engine thrust cannot be measured during flight, the ground experiments are conducted.

**Figure 6:**
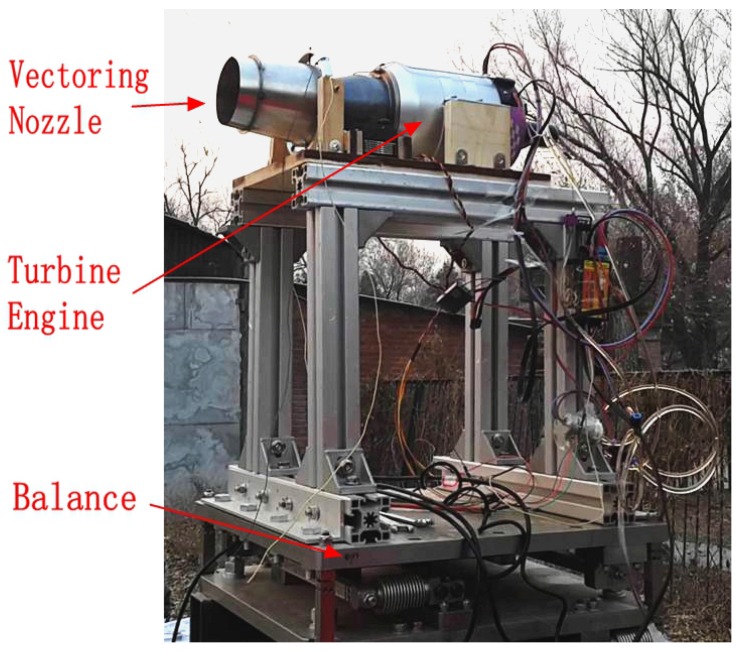
Engine thrust ground experiment platform.

Figure 6 shows the settings for engine thrust ground experiment. A turbine engine and a vectoring nozzle are fixed on a precise balance which can measure the triaxial forces. The detailed descriptions for the turbine engine ground experiment can be found at [24]. For the thrust experiment, the deflection angle of the vectoring nozzle is set to be zero, meaning the nozzle axis is aligned with the engine axis. During the experiment, the rotor speed is fixed at a constant value and the corresponding thrust is measured at the steady state. The rotor speed is then adjusted to different values and the thrust measurements are taken to build the model.

**Figure 7:**
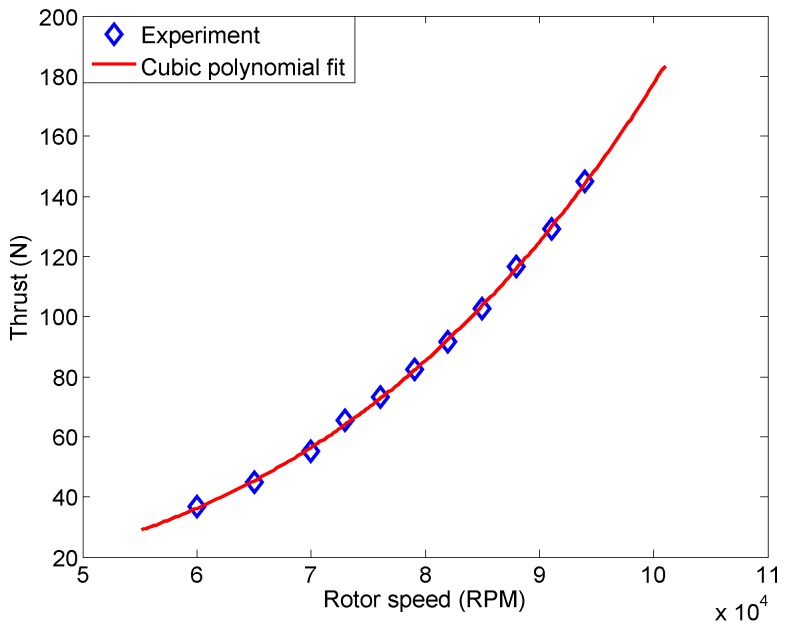
Relationship between the rotor speed and engine thrust in steady state conditions.

The thrust experiment results for a single P200 engine at steady state are shown in Figure 7 as the blue points for rotor speed ranges from 60,000 RPM to 94,000 RPM. A cubic polynomial function is used to fit the experimental data, and it gives us the red curve as shown in Figure 7. The mathematical expression for the cubic polynomial relationship is written as:

(5) $$F_{t}=439.61\Omega^{3}-499.46\Omega^{2}+293.67\Omega -55.26$$

where $F_{t}$ represents the engine thrust with the unit of Newton (N) and Ω represents the rotor speed with the unit of $10^{5}$ RPM. It can be seen that the polynomial function matches the experiment data closely. The mean of the regression residuals is $-1.2\times 10^{-4}$ N and the standard deviation of the residuals is 0.67 N.

The above polynomial relationship is validated by comparing the experimental thrust with the computed thrust (using Equation (5)). The comparison is shown in Table 2 for the rotor speeds of 96,000, 98,000 and 100,000 RPM which are not used in obtaining Equation (5). The relative error, designated $E_{F}$, is computed as:

(6) $$E_{F}=\frac{F_{tm}-F_{te}}{F_{te}}$$

where $F_{tm}$ is the engine thrust computed using the model, and $F_{te}$ is the engine thrust measured in the experiment. The close agreement between the measured and computed thrusts and the very small (0.3%) relative errors shown in Table 2 demonstrate high accuracy of the thrust model. Different experimental data (rather than the blue points shown in Figure 7) can be used to fit the cubic equation as long as the relative errors shown in Table 2 are within an acceptable range (0.5% used in this work). Detailed discussion about the cubic thrust model can be found in an earlier work by Tsinghua University [24].

**Figure 8:**
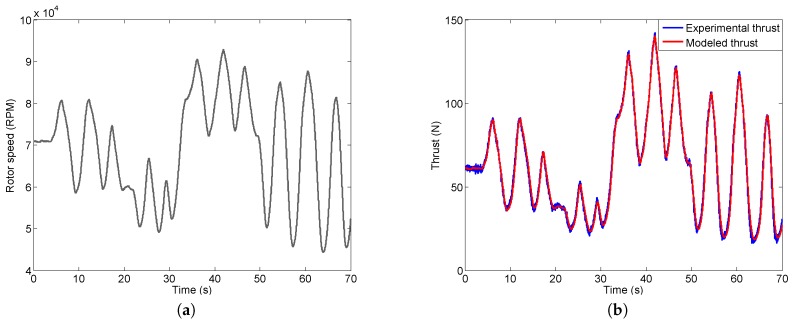
Thrust model validation using time-varying rotor speed. (**a**) Time-varying rotor speed; (**b**) Measured thrust and modeled thrust for time-varying rotor speed.

For real problems, the engines typically operate with time-varying rotor speed, especially during the takeoff and landing stages. Therefore, the polynomial thrust model needs to be validated by a dynamic thrust experiment as well. The time-varying rotor speed as shown in Figure 8a is used in the dynamic experiment. The range for this time-varying rotor speed is from 45,000 to 95,000 RPM which covers the rotor speed for UAV takeoff and landing. The corresponding thrust computed using the polynomial model is then compared with the thrust measured during the experiment in Figure 8b. A close match is observed which again confirms the accuracy and effectiveness of the thrust model.

As mentioned before, the engine thrust cannot be measured during flight, but the rotor speed can be measured by Hall sensor with very high accuracy. The measured rotor speed will serve as the input in the engine thrust model (Equation (5)) to compute the thrust.

### 4.2. Thrust Vector Model

During flight, the attitude of the UAV is controlled by the engine thrust vectoring system to ensure the stability of the UAV body (with the UAV body nose pointing up vertically). The thrust vectoring system generates triaxial moments by deflecting the vectoring nozzles. Consequently, the engine thrust is not exactly aligned with the engine axis due to the nozzle deflection. The engine thrust model (Equation (5)) developed in the previous section is used to compute the overall thrust. To obtain the vertical component of the overall thrust, the deflection angle of the engine thrust is needed.

We are not able to measure the deflection angle of the thrust directly when the UAV is flying. However, the deflection angle is controlled by the microcomputer and the command of the deflection angle can be recorded. The thrust vectoring system is well calibrated to ensure the thrust deflection angle is the same as the deflection command. It is validated by performing a thrust vectoring experiment on the ground.

**Figure 9:**
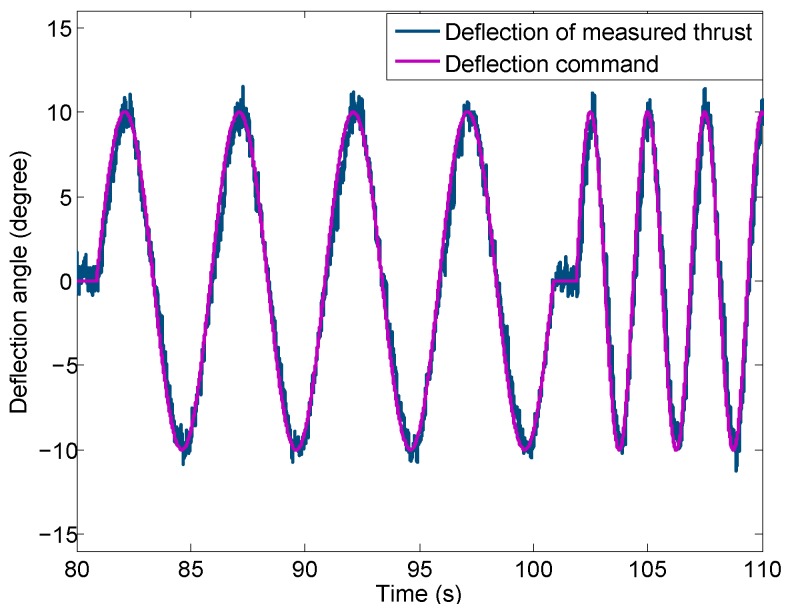
Comparison of the deflection angle command and the actual deflection angle of thrust.

The same experimental settings shown in Figure 6 are used. The engine rotor speed for this experiment is fixed at 84,140 RPM which corresponds to a total thrust of 100 N. Since the nozzle is axisymmetric, only the experimental results for yaw movement are presented. During the experiment, the sinusoidal deflection angle command with an amplitude of 10 degree (shown in Figure 9 as the purple curve) is used and the corresponding thrust vector is measured. The deflection angle of the thrust, designated $\alpha_{F}$, is calculated using Equation (7) as:

(7) $$\alpha_{F}=tan^{-1}\frac{F_{ty}^{b}}{F_{tx}^{b}}$$

where $F_{tx}^{b}$ and $F_{ty}^{b}$ are the measured thrusts in the *x* and *y* directions in the UAV body coordinate system. The UAV coordinate is defined as: the *x*-axis points out of the tailsitter nose, the *y*-axis points out the right wing and the *z*-axis points out the bottom of the fuselage.

The actual thrust deflection angle ($\alpha_{F}$) derived from the thrust vector measurement using Equation (7) is compared with the deflection command in Figure 9 using the blue curve. A close match between the two curves is observed. This suggests that the deflection command can be used as the actual thrust deflection angle with sufficient accuracy. Therefore, the thrust vector model can be written as:

(8) $$\left(\begin{array}{c} F_{tx}^{b}\\ F_{ty}^{b}\\ F_{tz}^{b} \end{array}\right)=\frac{F_{t}}{\sqrt{1+tan^{2}\alpha +tan^{2}\beta }}\left(\begin{array}{c} 1\\ tan\beta \\ tan\alpha \end{array}\right)$$

where *α* and *β* are the pitch and yaw deflection commands, $F_{t}$ is the total engine thrust which is estimated using engine thrust model described in the previous section, $F_{tx}^{b}$, $F_{ty}^{b}$ and $F_{tz}^{b}$ are the engine thrusts in the *x*, *y* and *z* directions respectively. Note that the *x*, *y* and *z* directions are in the UAV coordinate system, not in the inertial coordinate system.

By combining the engine thrust model (Equation (5)) and thrust vector model (Equation (8)), we are able to obtain the thrust vector using the measured rotor speed and the deflection commands. The coordinate transformation is again used to obtain the vertical thrust, which is indicated by $F_{vc}$.

Note that there are two engines in the propulsion system. During the flight, the two engines receive the same rotor speed command, which means they have the same overall thrust. But the deflection angles are controlled independently. Therefore, the vertical thrusts generated by the two engines, designated $F_{vc1}$ and $F_{vc2}$, need to be computed separately. The total vertical thrust of the engine system is the sum of $F_{vc1}$ and $F_{vc2}$:

(9) $$F_{vc}=F_{vc1}+F_{vc2}$$

Though the modeled results are of good quality, small modeling errors are inevitable. In addition, the measurement noise is also present. To incorporate these effects into the vertical thrust computation, we assume the error of the computed vertical thrust is white Gaussian noise. The genuine vertical thrust is thus expressed as:

(10) $$F_{v}=F_{vc}+w_{F}$$

where $F_{v}$ is the genuine vertical thrust, $F_{vc}$ is the computed vertical thrust, and $w_{F}$ is the white Gaussian noise for vertical thrust with the variance of $\sigma_{F}^{2}$.

## 5. Methodology for Acceleration Noise Suppression

By defining $\lambda =\frac{1}{m}$ in which *m* denotes the UAV mass, we can rewrite Equation (4) as:

(11) $$a_{m}=\lambda F_{v}+w_{m}$$

where $F_{v}$ is the vertical thrust force and $a_{m}=a+g+w_{m}$ represents the measurement of the IMU with $w_{m}$ is the vertical acceleration noise which is white Gaussian noise with a variance of $\sigma_{a_{m}}^{2}$. By combining with Equation (10), the above equation can be rewritten as:

(12) $$\begin{array}{cc} a_{m} & =\lambda \left(F_{vc}+w_{F}\right)+w_{m}\\ & =\lambda F_{vc}+\left(\lambda w_{F}+w_{m}\right) \end{array}$$

Since *λ* is a constant (the inverse of the UAV mass), $\lambda w_{F}$ is white Gaussian noise with the variance of $\lambda^{2}\sigma_{F}^{2}$.

We then define a new term, *w*, as:

(13) $$w=\lambda w_{F}+w_{m}$$

where *w* represents the overall noise which combines the measured acceleration noise and the thrust modeling error. Because both $\lambda w_{F}$ and $w_{m}$ are white Gaussian noise, *w* is white Gaussian noise as well. In addition, $\lambda w_{F}$ is independent of $w_{m}$ due to the independence between $w_{F}$ and $w_{m}$. According to the above analysis, *w* can be treated as white Gaussian noise with the variance, designated $\sigma^{2}$, written as:

(14) $$\sigma^{2}=\lambda^{2}\sigma_{F}^{2}+\sigma_{a_{m}}^{2}$$

We take *λ* as the state variable, $a_{m}$ as the observation variable and $F_{vc}$ as the input parameter, the discrete-time state space model can be written as:

(15) $$\left\{\begin{array}{c} \lambda (k+1)=\lambda (k)\\ a_{m}\left(k\right)=\lambda \left(k\right)F_{vc}\left(k\right)+w\left(k\right) \end{array}\right.$$

The standard Kalman filter can be used to estimate the *λ* by combining the computed thrust and measured acceleration information. The updating process is as follows:

(16) $$\left\{\begin{array}{c} K_{\lambda }\left(k\right)=P_{\lambda }\left(k-1\right)F_{vc}\left(k\right){\left[F_{vc}\left(k\right)P_{\lambda }\left(k-1\right)F_{vc}\left(K\right)+\sigma^{2}\left(k\right)\right]}^{-1}\\ P_{\lambda }\left(k\right)=\left[1-K_{\lambda }\left(k\right)F_{vc}\left(k\right)\right]P_{\lambda }\left(k-1\right)\\ \hat{\lambda }\left(k\right)=\hat{\lambda }\left(k-1\right)+K_{\lambda }\left(k\right)\left[a_{m}\left(k\right)-F_{vc}\left(k\right)\hat{\lambda }\left(k-1\right)\right] \end{array}\right.$$

where $\hat{\lambda }\left(k-1\right)$ and $\hat{\lambda }\left(k\right)$ are the estimates of *λ* at sampled data points k−1 and *k* respectively, $K_{\lambda }\left(k\right)$ is the Kalman filter gain, and $P_{\lambda }\left(k\right)$ is the variance of $\hat{\lambda }\left(k\right)$. Note that the variance of w(k), designated $\sigma^{2}\left(k\right)$, is estimated as follows in the calculation process:

(17) $$\sigma^{2}\left(k\right)={\hat{\lambda }}^{2}\left(k-1\right)\sigma_{F}^{2}+\sigma_{a_{m}}^{2}$$

Here we assume the variance of the measured acceleration noise during UAV takeoff and landing is the same as the acceleration variance when the engine rotor speed is at 85,000 RPM. This is because that the rotor speed of 85,000 RPM is very close to speed of the engines during takeoff and landing. Therefore, $\sigma_{a_{m}}=0.351$ (from Table 1) is used in this work. The variance of vertical thrust error is assumed to be $\sigma_{F}^{2}=10$ N${}^{2}$.

With the estimated *λ* and calculated vertical thrust $F_{vc}$, vertical acceleration can be obtained as:

(18) $$a_{c}\left(k\right)=\hat{\lambda }\left(k\right)F_{vc}\left(k\right)$$

where $a_{c}\left(k\right)$ is the calculated acceleration at sampled data point *k*. By combing with Equation (10), the above equation can be rewritten as:

(19) $$\begin{array}{cc} a_{c}\left(k\right) & =\hat{\lambda }\left(k\right)\left[F_{v}\left(k\right)-w_{F}\left(k\right)\right]\\ & =\hat{\lambda }\left(k\right)F_{v}\left(k\right)-\hat{\lambda }\left(k\right)w_{F}\left(k\right) \end{array}$$

We then investigate the expectation of $a_{c}\left(k\right)$ as:

(20) $$\begin{array}{cc} E\{a_{c}\left(k\right)\} & =E\left\{\hat{\lambda }\left(k\right)F_{v}\left(k\right)\right\}-E\left\{\hat{\lambda }\left(k\right)w_{F}\left(k\right)\right\}\\ & =\lambda F_{v}\left(k\right)-\rho_{\hat{\lambda }\left(k\right),w_{F}\left(k\right)}\sigma_{\hat{\lambda }\left(k\right)}\sigma_{F} \end{array}$$

where $\rho_{\hat{\lambda }\left(k\right),w_{F}\left(k\right)}$ represents the correlation coefficient between $\hat{\lambda }\left(k\right)$ and $w_{F}\left(k\right)$, and the derivation of $\rho_{\hat{\lambda }\left(k\right),w_{F}\left(k\right)}$ is shown in Equation (A1). Note that the absolute value of the correlation coefficient does not exceed 1. Because $\sigma_{\hat{\lambda }\left(k\right)}$ approaches zero as *k* tends to infinity, the expectation of $a_{c}\left(k\right)$ approaches the genuine vertical acceleration, $\lambda F_{v}\left(k\right)$, as *k* tends to infinity according to Equation (20). Therefore $a_{c}\left(k\right)$ is the asymptotically unbiased estimate of the vertical acceleration.

According to Equation (19), the variance of $a_{c}\left(k\right)$ is then given by:

(21) $$\begin{array}{cc} \sigma_{a_{c}}^{2}\left(k\right) & =F_{v}^{2}\left(k\right)\sigma_{\hat{\lambda }\left(k\right)}^{2}+{\hat{\lambda }\left(k\right)}^{2}\sigma_{F}^{2}-2\rho_{\hat{\lambda }\left(k\right),w_{F}\left(k\right)}\hat{\lambda }\left(k\right)F_{v}\left(k\right)\sigma_{\hat{\lambda }\left(k\right)}\sigma_{F} \end{array}$$

To obtain the minimum variance estimate of the vertical acceleration, the calculated acceleration $a_{c}\left(k\right)$ and the measured acceleration $a_{m}\left(k\right)$ are fused as :

(22) $$a_{f}\left(k\right)=\eta (k)a_{c}\left(k\right)+\left[1-\eta \left(k\right)\right]a_{m}\left(k\right)$$

where $a_{f}\left(k\right)$ is the acceleration fusion result, η(k) is the weighting coefficient with 0<η(k)<1. It is obvious that $a_{f}\left(k\right)$ is asymptotically unbiased due to the asymptotically unbiasedness of $a_{c}\left(k\right)$ and the unbiasedness of $a_{m}\left(k\right)$. The variance of $a_{f}\left(k\right)$ is given by:

(23) $$\sigma_{a_{f}\left(k\right)}^{2}={\eta (k)}^{2}\sigma_{a_{c}\left(k\right)}^{2}+{\left[1-\eta \left(k\right)\right]}^{2}\sigma_{a_{m}}^{2}+2\eta (k)\left[1-\eta \left(k\right)\right]\rho_{a_{c}\left(k\right),a_{m}\left(k\right)}\sigma_{a_{c}\left(k\right)}\sigma_{a_{m}}$$

where $\rho_{a_{c}\left(k\right),a_{m}\left(k\right)}$ is the correlation coefficient between $a_{c}\left(k\right)$ and $a_{m}\left(k\right)$ which is derived in Equation (A2). The variable η(k) is adopted as follows to achieve the minimum variance estimate:

(24) $$\eta \left(k\right)=\frac{\sigma_{a_{m}}^{2}-\rho_{a_{c}\left(k\right),a_{m}\left(k\right)}\sigma_{a_{c}\left(k\right)}\sigma_{a_{m}}}{\sigma_{a_{c}\left(k\right)}^{2}+\sigma_{a_{m}}^{2}-2\rho_{a_{c}\left(k\right),a_{m}\left(k\right)}\sigma_{a_{c}\left(k\right)}\sigma_{a_{m}}}$$

**Figure 10:**
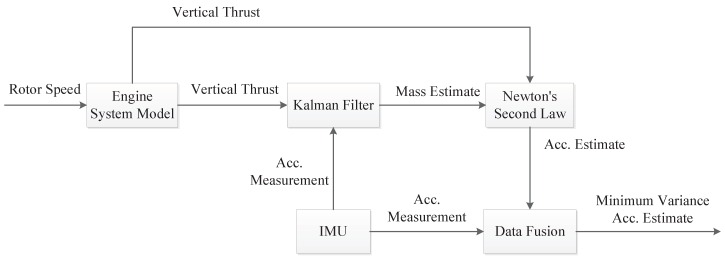
Flowchart of the noise reduction procedure developed in this work.

The flowchart of the noise reduction procedure developed in this work is shown in Figure 10. At first, the engine system model (including engine thrust model and thrust vector model in Section 4) needs to be built. It is used to obtain the UAV vertical thrust when it is in flight. Then the Kalman filter (Equation (16)) is applied to estimate UAV mass by combining the information of engine vertical thrust and the IMU measured acceleration. The estimated UAV mass can then be used together with the vertical thrust to compute acceleration using Newton’s Second Law. A data fusion step (Equation (22)) is finally applied to obtain the minimum variance estimate of the UAV acceleration. This overall procedure can be used for any type of UAVs for noise reduction purpose. The only change will be in the engine system model if the method is applied to different UAV platforms.

## 6. Experiment Result

**Figure 11:**
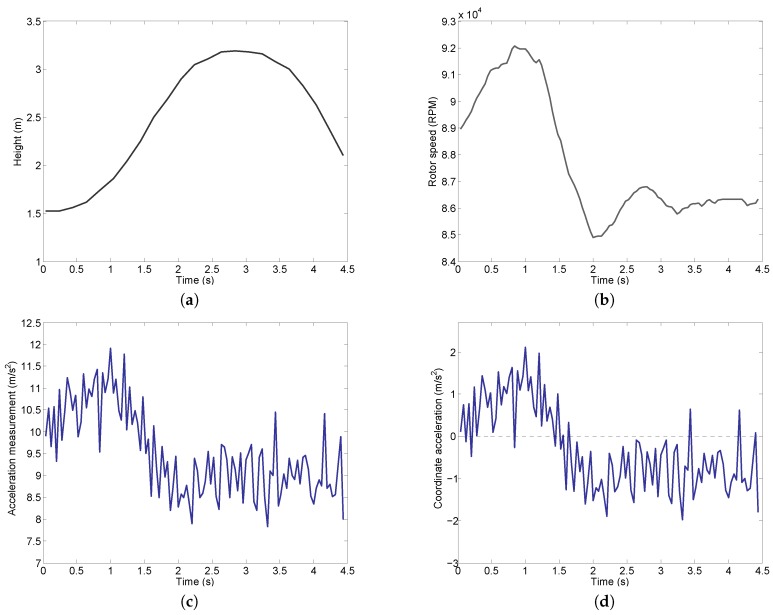
Experiment results. (**a**) Experiment height measurement; (**b**) Experiment rotor speed; (**c**) Experiment acceleration measurement; (**d**) Coordinate acceleration (computed from Figure 11c).

To assess the performance of the new method for UAV vertical acceleration noise suppression, a vertical takeoff experiment is conducted. The data record began at the time when the UAV has positive (upward) elevation change which is indicated by the height measurement from the laser range finder as shown in Figure 11a. The UAV ascended from the initial position of about 1.5 m to around 3.2 m at t=2.8 s. This corresponds to a maximum height gain of about 1.7 m. After reaching the highest point, the UAV started to descend, though it had not returned to its original position for the period that we measured. The rotor speed measured during the experiment is shown in Figure 11b. As can be seen, the rotor speed increased for t<1 s, and dropped quickly during 1<t<2 s. The rotor speed reached a stable value around 86,000 RPM eventually. The corresponding vertical acceleration measurement from IMU is shown in Figure 11c. The same as in Figure 3, the acceleration measurement is heavily polluted by the noise, though the similar trend with the rotor speed is observed.

We then subtract the gravitational acceleration from the IMU measured acceleration shown in Figure 11c. This gives us the coordinate acceleration (rate of change of velocity) which is displayed in Figure 11d. It can be clearly seen that this UAV experiment includes both the upward acceleration period (positive coordinate acceleration) and downward acceleration period (negative coordinate acceleration). Therefore, it is sufficient to validate the noise reduction method developed in this work.

**Figure 12:**
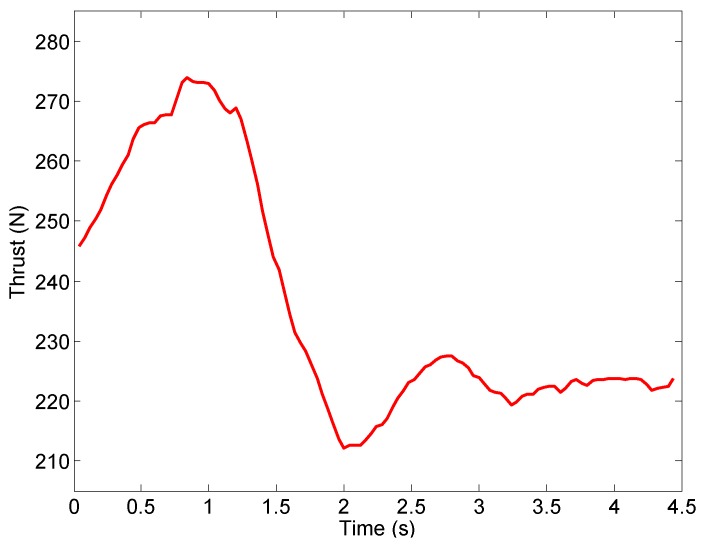
Calculated vertical thrust.

**Figure 13:**
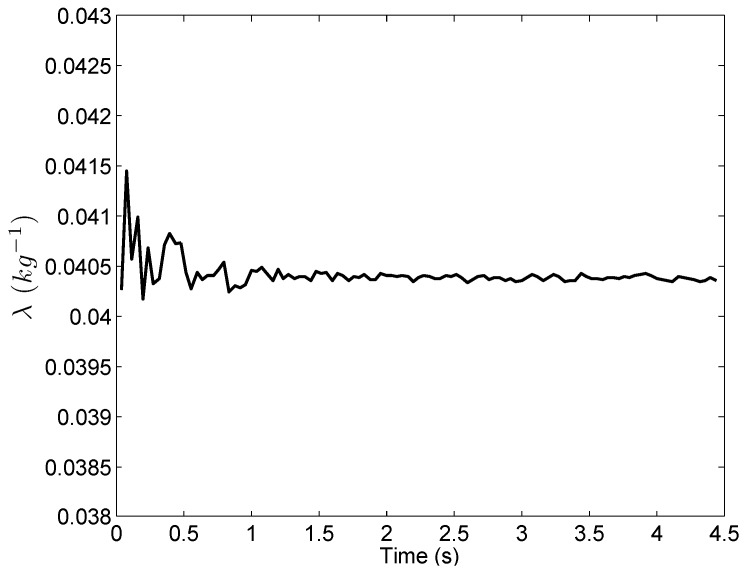
Estimate of *λ*.

We apply the engine thrust model and thrust vector model developed in Section 3 to the experimental data. The calculated vertical thrust is shown in Figure 12. It is then used to estimate *λ* using the Kalman filter (Equation (16)). As shown in Figure 13, the value of *λ* quickly converges to 0.0404 kg${}^{-1}$, which means that the mass of the UAV (1/*λ*) is about 24.75 kg. Note that, the variable *λ* is unknown during flight, though it can be considered as a constant within a short period of time (for example, during the takeoff and landing stages). This is because that the UAV mass is impacted by the fuel consumption, which is unknown when the UAV is flying. Therefore, the Kalman filter (Equation (16)) is required to estimate *λ*.

**Figure 14:**
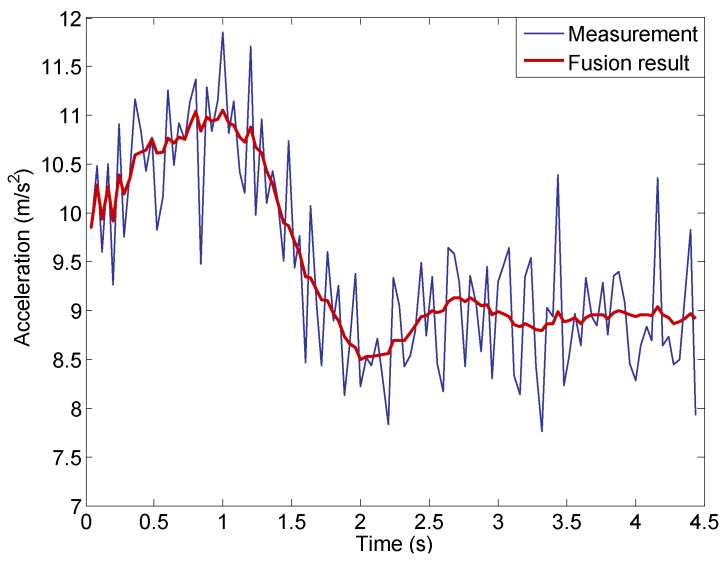
Acceleration fusion result.

**Figure 15:**
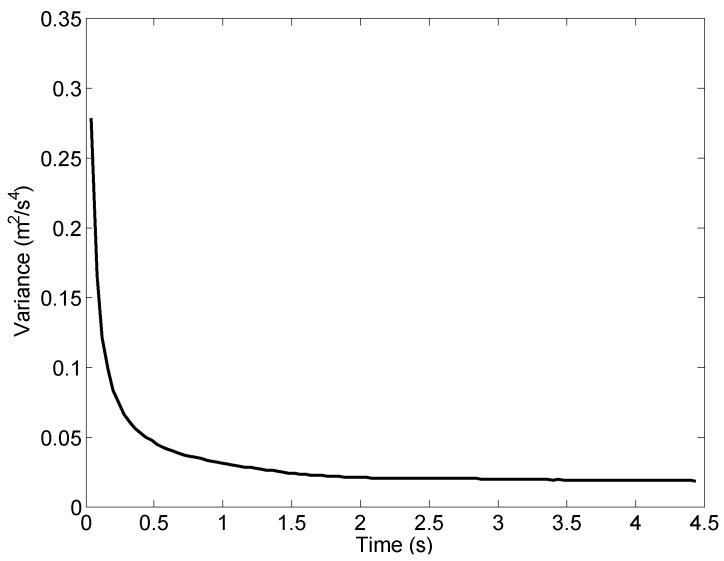
Variance of computed acceleration.

The final acceleration fusion result is shown in Figure 14 as the red line. For comparison purpose, the measured vertical acceleration is shown again in Figure 14. It can be seen clearly that the acceleration fusion result suppresses the noise in the measured acceleration and meanwhile preserves the trend in measurements. To quantify that, we calculate the variance of the acceleration computed with data fusion method using Equation (23). The variance is shown in Figure 15. Initially the variance is of similar magnitude as the variance of the measured acceleration (as shown in Table 1). It drops quickly as more data is used in the fusion calculation and becomes stable at a value close to 0.02 m${}^{2}$/s${}^{4}$ after about 2 s.

For the purpose of comparison, an infinite impulse response (IIR) lowpass filter developed in [9] and the Kalman filter developed in [13] are also used to denoise the measured acceleration. The parameters of the IIR filter and the Kalman filter are adopted to achieve similar denoising performance to the data fusion method developed in this work.

The IIR lowpass filter is shown as follows:

(25) $$\begin{array}{cc} a_{l}\left(k\right)= & b_{0}a_{m}\left(k\right)+b_{1}a_{m}\left(k-1\right)+b_{2}a_{m}\left(k-2\right)+b_{3}a_{m}\left(k-3\right)+b_{4}a_{m}\left(k-4\right)+b_{5}a_{m}\left(k-5\right)\\ & -c_{1}a_{l}\left(k-1\right)-c_{2}a_{l}\left(k-2\right)-c_{3}a_{l}\left(k-3\right)-c_{4}a_{l}\left(k-4\right)-c_{5}a_{l}\left(k-5\right) \end{array}$$

where $a_{l}\left(k\right)$ and $a_{m}\left(k\right)$ are the filtered and measured accelerations for sampled data point *k*, respectively. The coefficients used here are:

$$\begin{array}{cc} & b_{0}=0.0013,b_{1}=0.0064,b_{2}=0.0128,b_{3}=0.0128,b_{4}=0.0064,b_{5}=0.0013\\ & c_{1}=-2.9754,c_{2}=3.8060,c_{3}=-2.5453,c_{4}=0.8811,c_{5}=-0.1254 \end{array}$$

The state space model used in the Kalman filter developed in [13] is shown as:

(26) $$\left\{\begin{array}{c} a_{s}\left(k+1\right)=a_{s}\left(k\right)+w_{s}\left(k\right)\\ a_{m}\left(k\right)=a_{s}\left(k\right)+w_{m}\left(k\right) \end{array}\right.$$

where $a_{k}\left(k\right)$ and $a_{k}\left(k+1\right)$ are the acceleration states at sampled point *k* and k+1, $w_{s}\left(k\right)$ is the process noise, $a_{m}\left(k\right)$ is the observed acceleration with observation noise indicated by $w_{m}\left(k\right)$. Note that $w_{s}\left(k\right)$ and $w_{m}\left(k\right)$ are mutually independent white Gaussian noise. Their variances used here are adopted as $\sigma_{w_{s}}^{2}=0.01$ and $\sigma_{w_{m}}^{2}=0.351$, respectively.

**Figure 16:**
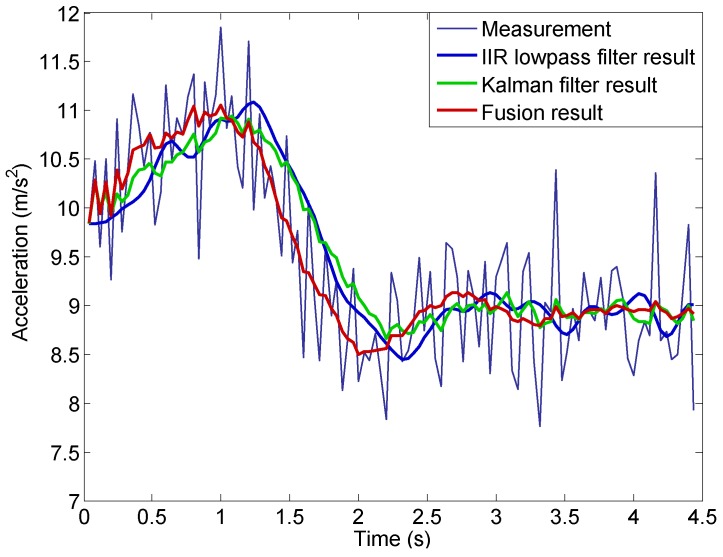
Acceleration fusion result compared with the IIR lowpass filter and Kalman filter results.

The comparison is shown in Figure 16. At a very early time value (t<0.2 s), the results using the Kalman filter and the new data fusion method are very close. As more data is included into the calculation, they start to deviate and an obvious time delay is seen in the Kalman filter result for t<2.5 s. In contrast, the lowpass result differs from the fusion result from the very beginning, and the time delay in the lowpass result is very obvious as well. At the late stage (t>2.5 s), when the acceleration becomes relatively stable (due to stable engine rotor speed shown in Figure 11b), the results using the three approaches become close.

**Figure 17:**
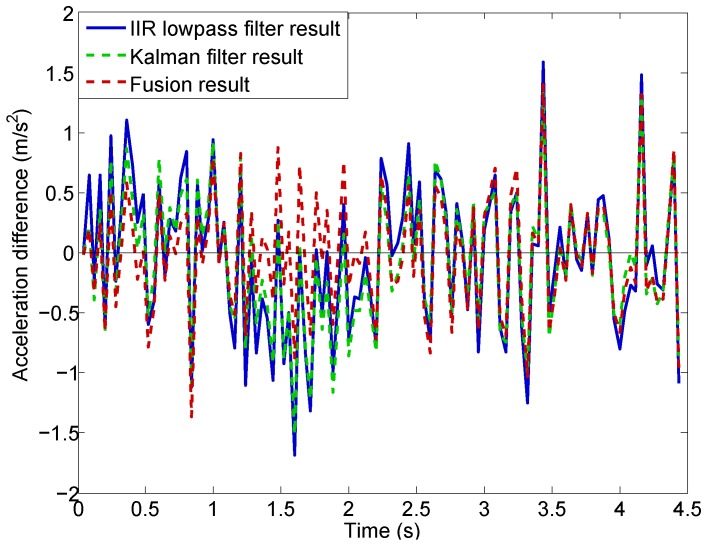
Acceleration differences.

The difference between each of the three computed accelerations and the measured acceleration, designated $a_{d}$, is computed as:

(27) $$a_{d}=a_{c}-a_{m}$$

where $a_{c}$ is the computed acceleration, and $a_{m}$ is the measured acceleration. The acceleration differences are shown in Figure 17. Dashed lines are used for Kalman filter and data fusion results for better visualization. We can see that the acceleration differences are of high amplitude due to the significant noise in the measured acceleration.

**Figure 18:**
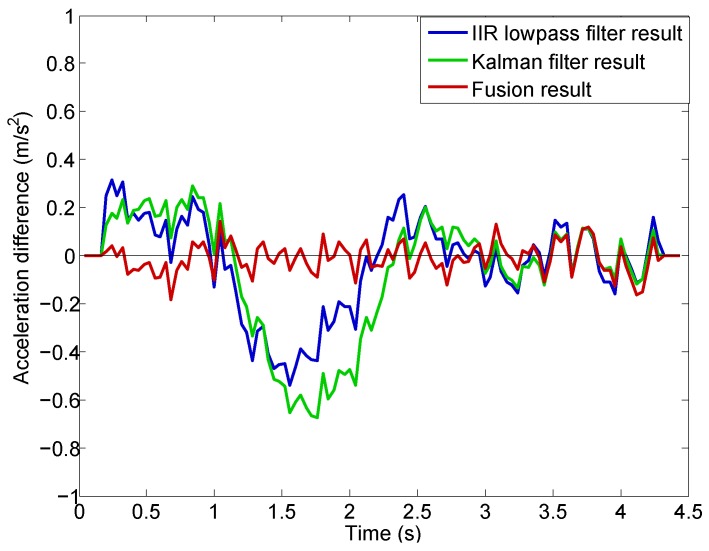
Low frequency component of acceleration differences.

A nine-order mean filter is used to extract the low frequency component of the acceleration differences shown in Figure 17. The nine-order mean filter is expressed as:

(28) $${\bar{a}}_{d}\left(k\right)=\frac{1}{9}\sum_{i=k-4}^{k+4}a_{d}\left(i\right),\;\mathrm{for}\;4<k<N-4$$

where ${\bar{a}}_{d}\left(k\right)$ is the low frequency component result at sampled data point *k*. The results are shown in Figure 18, from which we can clearly see the difference between the new method and the two existing methods. The lowpass filter and Kalman filter provide similar results and both of them have apparent non-zero values in their low frequency components (greater than zero for t<0.9 s and less than zero during 1<t<2.2 s). This is because of the time delay introduced by the filtering process. The time delay is due to the weighting of the past time acceleration measurements in the filtered results in both the lowpass and Kalman filters. In contrast, the results using the new method closely distribute around zero without any obvious non-zero trends. This is because that the new method uses the past time information only for the estimation of the variable *λ*. As shown in Figure 13, the *λ* converges very quickly to a constant value during the takeoff stage. Therefore, the new method does not introduce any time delay in acceleration computation.

Note that the time delay can be reduced for lowpass filter and Kalman filter by adjusting their parameters, but the noise suppression performance of the two methods will be severely degraded. Compared with the lowpass filter and Kalman filter, the newly developed method aided with the thrust information have much better real time performance with strong ability of noise suppression.

## 7. Conclusions

In this work, we developed a novel method to reduce the noise in acceleration measurement for a thrust-vectored VTOL UAV. The method combines the information in engine thrust with the information in the measured acceleration. To do that, a Kalman filter is first applied to estimate the UAV mass, which is used to compute acceleration together with the vertical thrust. This estimated acceleration is further fused with the measured acceleration to obtain the minimum variance acceleration estimate.

To test the effectiveness of the newly developed approach, a vertical takeoff experiment for the UAV was performed. The new method was used to compute the vertical acceleration using the data collected from the experiment. The results demonstrated that the new method has very good performance in terms of noise reduction. The variance of the vertical acceleration is reduced by about 95%. In addition, the acceleration calculated using the new approach shows no time delay, which enables its usage in acceleration feedback control for UAV takeoff and landing (or for any other situations with fast-changing accelerations). In contrast, two existing approaches based on IIR and Kalman filter suffer from significant time delay which limits their usability for real problems, although they can achieve similar denoising performance.

In addition, we developed numerical models for engine thrust and thrust vector which are important components in the entire noise suppression procedure. A polynomial function is used to characterize the relationship between the rotor speed and the engine thrust. Though the model was built based on steady-state ground experiment, it is able to predict accurately the thrust in dynamic condition (with time-varying rotor speed). The thrust deflection angle was found to be consistent with the deflection angle command, which is then used to compute the engine thrust in the vertical direction.

We analyzed the statistical characteristics of the acceleration noise. It was found that the amplitude of the noise grows with the increasing rotor speed. We also demonstrated that the acceleration noise can be treated as white Gaussian noise based on the autocorrelation and histogram of the sampled noise data.

The noise suppression method developed here can be readily applied into the acceleration feedback motion control for VTOL UAVs during a critical period, for example, takeoff and landing stages. Improved precision in motion control is expected, which will be tested in future work.

## Figures and Tables

**Table 1:** Mean and variance of acceleration measurement.

Engine States	Mean (m/s${}^{2}$)	Variance (m${}^{2}$/s${}^{4}$)
Before start	9.801	0.00022
Idle speed (33,000 RPM)	9.792	0.193
Rotor speed (85,000 RPM)	9.797	0.351

**Table 2:** Comparison of the experimental thrust and the modeled thrust.

Rotor Speed (RPM)	96,000	98,000	100,000
Experimental thrust (N)	154.8	167.3	177.1
Computed thrust (N)	154.6	166.8	177.4
Relative error (%)	−0.13	−0.30	0.17

## Abbreviations

The following abbreviations are used in this manuscript:

UAV	Unmanned aerial vehicle
VTOL	Vertical takeoff and landing
IMU	Inertial measurement unit
RPM	Revolutions per minute
IIR	Infinite impulse response

## Appendix A

(A1) $$\begin{array}{cc} \rho_{\hat{\lambda }\left(k\right),w_{F}\left(k\right)} & =E\left\{w_{F}\left(k\right)\left(\hat{\lambda }\left(k\right)-\lambda \right)\right\}/\left(\sigma_{\hat{\lambda }\left(k\right)}\sigma_{F}\right)\\ & =E\left\{w_{F}\left(k\right)\left[\hat{\lambda }\left(k-1\right)+K_{\lambda }\left(k\right)\left(a_{m}\left(k\right)-F_{vc}\left(k\right)\hat{\lambda }\left(k-1\right)\right)-\lambda \right]\right\}/\left(\sigma_{\hat{\lambda }\left(k\right)}\sigma_{F}\right)\\ & =E\left\{w_{F}\left(k\right)\left[\hat{\lambda }\left(k-1\right)+K_{\lambda }\left(k\right)\left(\lambda F_{vc}\left(k\right)+\lambda w_{F}\left(k\right)+w_{m}\left(k\right)-F_{vc}\left(k\right)\hat{\lambda }\left(k-1\right)\right)-\lambda \right]\right\}/\left(\sigma_{\hat{\lambda }\left(k\right)}\sigma_{F}\right)\\ & =E\left\{w_{F}\left(k\right)\left[\left(\lambda -\hat{\lambda }\left(k-1\right)\right)\left(K_{\lambda }\left(k\right)F_{vc}\left(k\right)-1\right)+w_{F}\left(k\right)K_{\lambda }\left(k\right)\left(\lambda w_{F}\left(k\right)+w_{m}\left(k\right)\right)\right]\right\}/\left(\sigma_{\hat{\lambda }\left(k\right)}\sigma_{F}\right)\\ & =E\left\{K_{\lambda }\left(k\right)w_{F}\left(k\right)\left[\lambda w_{F}\left(k\right)+w_{m}\left(k\right)\right]\right\}/\left(\sigma_{\hat{\lambda }\left(k\right)}\sigma_{F}\right)\\ & =K_{\lambda }\left(k\right)\lambda \sigma_{F}/\sigma_{\hat{\lambda }\left(k\right)} \end{array}$$

(A2) $$\begin{array}{cc} \rho_{a_{c}\left(k\right),a_{m}\left(k\right)} & =E\left\{w_{m}\left(k\right)\left[\hat{\lambda }\left(k\right)w_{F}\left(k\right)+\hat{\lambda }\left(k\right)F_{v}\left(k\right)-\lambda F_{v}\left(k\right)\right]\right\}/\left(\sigma_{a_{c}\left(k\right)}\sigma_{a_{m}}\right)\\ & =E\left\{\hat{\lambda }\left(k\right)w_{m}\left(k\right)w_{F}\left(k\right)+w_{m}\left(k\right)\left(\hat{\lambda }\left(k\right)-\lambda \right)F_{v}\left(k\right)\right\}/\left(\sigma_{a_{c}\left(k\right)}\sigma_{a_{m}}\right)\\ & =F_{v}\left(k\right)E\left\{w_{m}\left(k\right)\left[\hat{\lambda }\left(k\right)-\lambda \right]\right\}/\left(\sigma_{a_{c}\left(k\right)}\sigma_{a_{m}}\right)\\ & =F_{v}\left(k\right)E\left\{w_{m}\left(k\right)\left[\hat{\lambda }\left(k-1\right)+K_{\lambda }\left(k\right)\left(a_{m}\left(k\right)-F_{vc}\left(k\right)\hat{\lambda }\left(k-1\right)\right)-\lambda \right]\right\}/\left(\sigma_{a_{c}\left(k\right)}\sigma_{a_{m}}\right)\\ & =F_{v}\left(k\right)E\left\{w_{m}\left(k\right)\left[\left(\lambda -\hat{\lambda }\left(k-1\right)\right)\left(K_{\lambda }\left(k\right)F_{vc}\left(k\right)-1\right)+K_{\lambda }\left(k\right)w_{m}\left(k\right)+K_{\lambda }\lambda w_{F}\left(k\right)\right]\right\}/\left(\sigma_{a_{c}\left(k\right)}\sigma_{a_{m}}\right)\\ & =F_{v}\left(k\right)K_{\lambda }\sigma_{a_{m}}/\sigma_{a_{c}\left(k\right)} \end{array}$$

## References

[1] Johnson E.N., Turbe M.A. (2006). Modeling, control, and flight testing of a small-ducted fan aircraft. J. Guid. Control Dyn..

[2] Kubo D., Suzuki S. (2008). Tail-sitter vertical takeoff and landing unmanned aerial vehicle: Transitional flight analysis. J. Aircr..

[3] Tomic T., Schmid K., Lutz P., Domel A., Kassecker M., Mair E., Grixa I.L., Ruess E., Suppa M., Burschka D. (2012). Toward a fully autonomous UAV: Research platform for indoor and outdoor urban search and rescue. IEEE Robot. Autom. Mag..

[4] Lei X., Li J. (2012). An adaptive altitude information fusion method for autonomous landing processes of small unmanned aerial rotorcraft. Sensors.

[5] Peddle I.K. (2008). Acceleration Based Manoeuvre Flight Control System for Unmanned Aerial Vehicles.

[6] Boyle D.P., Chamitoff G.E. (1999). Autonomous maneuver tracking for self-piloted vehicles. J. Guid. Control Dyn..

[7] Wang X. (2013). Takeoff/landing control based on acceleration measurements for VTOL aircraft. J. Frankl. Inst..

[8] Gan Y., Sui L., Wu J., Wang B., Zhang Q., Xiao G. (2014). An EMD threshold de-noising method for inertial sensors. Measurement.

[9] Sun F., Sun W. (2010). Mooring alignment for marine SINS using the digital filter. Measurement.

[10] Parks T.W., Burrus C.S. (1987). Digital Filter Design.

[11] Lu S., Xie L., Chen J. (2009). New techniques for initial alignment of strapdown inertial navigation system. J. Frankl. Inst..

[12] El-Sheimy N., Nassar S., Noureldin A. (2004). Wavelet de-noising for IMU alignment. IEEE Aerosp. Electron. Syst. Mag..

[13] Kownacki C. (2011). Optimization approach to adapt Kalman filters for the real-time application of accelerometer and gyroscope signals’ filtering. Dig. Signal Process..

[14] Hebbale K.V., Ghoneim Y.A. A speed and acceleration estimation algorithm for powertrain control. Proceedings of the 1991 American Control Conference.

[15] Abellanosa C.B., Lugpatan R.P.J., Pascua D.A.D. (2016). Position Estimation using Inertial Measurement Unit (IMU) on a Quadcopter in an Enclosed Environment. Int. J. Comput. Commun. Instrum. Eng..

[16] Quadri S.A., Sidek O., Bin Abdullah A. (2014). A Study of State Estimation Algorithms in an OktoKopter. Int. J. u- e-Serv. Sci. Technol..

[17] Edu I.R., Adochiei F.C., Grigorie T.L., Botez R.M. (2015). Tuning of a Wavelet Filter for Miniature Accelerometers Denoising based Joint Symbolic Dynamics (JSD) Method. INCAS Bull..

[18] Nebylov A., Sukrit S., Arifuddin F. Perspectives for Development of an Autonomous and Intelligent WIG-Craft and Its Peculiar Control Problems. Proceedings of the 2009 IFAC “AGNFC” Workshop Proceedings.

[19] Li H., Wu L., Li Y., Li C. Identification of turbine engine dynamics with the governor in the loop. Proceedings of the 2016 IEEE International Conference on Systems, Man, and Cybernetics (SMC).

[20] Stone R.H., Anderson P., Hutchison C., Tsai A., Gibbens P., Wong K.C. (2008). Flight testing of the t-wing tail-sitter unmanned air vehicle. J. Aircr..

[21] Beach J.M., Argyle M.E., McLain T.W., Beard R.W., Morris S. Tailsitter heading estimation using a magnetometer. Proceedings of the 2014 American Control Conference.

[22] Woodman O.J. (2007). An Introduction to Inertial Navigation.

[23] Titterton D., Weston J. (2004). Strapdown Inertial Navigation Technology.

[24] Yang J., Zhu J. Dynamic modelling of a small scale turbojet engine. Proceedings of the 2015 European Control Conference (ECC).

